# A maternal meal affects clinical Doppler parameters in the fetal middle cerebral artery

**DOI:** 10.1371/journal.pone.0209990

**Published:** 2018-12-31

**Authors:** Gun Lisbet Opheim, Manuela Zucknick, Tore Henriksen, Guttorm Haugen

**Affiliations:** 1 Department of Fetal Medicine, Oslo University Hospital—Rikshospitalet, Oslo, Norway; 2 Norwegian Advisory Unit on Woman`s Health, Oslo University Hospital—Rikshospitalet, Oslo, Norway; 3 Institute of Clinical Medicine, University of Oslo, Oslo, Norway; 4 Oslo Centre for Biostatistics and Epidemiology, Department of Biostatistics, Institute of Basic Medical Sciences, University of Oslo, Oslo, Norway; 5 Department of Obstetrics, Oslo University Hospital–Rikshospitalet, Oslo, Norway; Ehime University Graduate School of Medicine, JAPAN

## Abstract

**Introduction:**

Middle cerebral artery (MCA) and umbilical artery (UA) Doppler blood flow pulsatility indices (PIs) and MCA peak systolic velocity (PSV) are essential variables for clinically evaluating fetal well-being. Here we examined how a maternal meal influenced these Doppler blood flow velocity variables.

**Methods:**

This prospective cohort study included 89 healthy Caucasian women with normal singleton pregnancies (median age, 32 years). Measurements were performed at gestational weeks 30 and 36, representing the start and near the end of the energy-depositing period. Measured variables included the MCA-PI, UA-PI, fetal heart rate (FHR) and MCA-PSV. The cerebroplacental ratio (CPR) was calculated as the ratio of MCA-PI to UA-PI. The first examination was performed in the fasting state at 08:30 a.m. Then participants ate a standard breakfast (approximate caloric intake, 400kcal), and the examination was repeated ~105 min after the meal.

**Results:**

Without adjustment for FHR, fetal MCA-PI decreased after the meal at week 30 (‒0.115; p = 0.012) and week 36 (‒0.255; p < 0.001). All PI values were negatively correlated with FHR. After adjustment for FHR, MCA-PI still decreased after the meal at week 30 (‒0.087; p = 0.044) and week 36 (‒0.194; p < 0.001). The difference between the two gestational weeks was non-significant (p = 0.075). UA-PI values did not significantly change at week 30 (p = 0.253) or week 36 (p = 0.920). CPR revealed significant postprandial decreases of −0.17 at week 30 (p = 0.006) and −0.22 at week 36 (p = 0.001). Compared to fasting values, MCA-PSV was significantly higher after food intake: +3.9 cm/s at week 30 (p < 0.001) and +5.9 cm/s at week 36 (p < 0.001).

**Conclusion:**

In gestational weeks 30 and 36, we observed a postprandial influence that was apparently specific to fetal cerebral blood flow.

## Introduction

Fetal nutrition depends on many variables, including maternal body composition and maternal nutritional intake, placental transfer and metabolism of nutritional substances, and uteroplacental and umbilicoplacental blood flow. Moreover, variations in regional blood flow determine how nutritional substances are distributed within the fetal body.

In animal models a decrease of umbilical venous oxygen content to below 50% of normal prompts the initiation of several circulatory changes in the fetus [1]. Middle cerebral artery (MCA) Doppler flow velocity indices provide information regarding fetal adaptation to reduced oxygen supply [2]. A decreased MCA pulsatility index (PI) suggests a compensatory reduction of vascular resistance to the fetal brain, known as circulatory centralization or the “brain-sparing effect” [3]. This measurement is used for clinical evaluation of pregnancies complicated by late fetal growth restriction [4], but is considered to be of minor importance when evaluating growth restriction early in pregnancy [5].

Umbilical artery (UA) PI is used to evaluate placental functional capacity. High resistance in the UA is associated with placental compromise and intrauterine growth restriction [6]. Dividing MCA-PI by UA-PI yields the cerebroplacental ratio (CPR), which quantifies the redistribution of cardiac output [7]. The CPR reflects both placental status and fetal response, and is reportedly to be more sensitive to perinatal outcome than the separate variables [8] [9]. Low CPR values are significantly and independently associated with adverse pregnancy outcomes [10]. Peak systolic velocity (PSV) is another clinically important Doppler blood flow variable in the MCA. In fetal anemia, MCA-PSV increases as blood viscosity declines due to the reduction of red blood cell concentration [11].

Our group recently conducted a study in which 105 participants were examined at gestational weeks 30–32. We found that maternal non-physiological glucose loading induced a reduction in MCA-PI values, indicating reduced fetal cerebral vascular resistance irrespective of fetal size. We did not observe any corresponding postprandial change in UA-PI [12]. Our results indicated that the fetus responds to a short-term increase in nutrient supply by increasing blood flow to the brain. However, in terms of redistribution, it is not yet known how the fetus responds to regular maternal food intake in the context of a normal pregnancy.

In our present study, we aimed to observe how a standard breakfast meal (SBM) impacted fetal cerebral and umbilical Doppler blood flow velocity variables among healthy women with normal pregnancies in the third trimester of gestation. Examinations were performed at gestational weeks 30 and 36, which represent the beginning and near the end of the important energy-depositing phase of fetal life, during which maternal nutritional intake and status may be particularly influential.

## Material and methods

### Participants

137 women of Caucasian origin with singleton low-risk pregnancies were recruited during the routine 18–20 week ultrasonographic examination at our hospital. Exclusion criteria were maternal diseases known to affect pregnancy (e.g., renal diseases, diabetes or hypertension), serious complications during a previous pregnancy (e.g., pre-eclampsia or gestational diabetes), medication with an overt potential to affect fetoplacental and fetal circulation (e.g., antihypertensive drugs), fetal malformations detected by ultrasound or by invasive tests, food intolerance or food allergy and earlier participation in the project.

Of the 137 recruited participants, seven were excluded. The reasons for exclusion were severe growth restriction diagnosed in gestational week 30 (n = 1), trisomy 21 (n = 1), malformations (n = 1), and birth before gestational week 36 (n = 4). One participant developed gestational diabetes between gestational weeks 30 and 36 and was retained in the study; her data did not influence the results. An additional 41 women were excluded from the main analyses because one or more of the MCA-PI or UA-PI measurements were missing due to fetal movements or head position too deep in the pelvic cavity. Finally, a total of 89 women were included in the present study.

### Ultrasonographic evaluation

For this prospective cohort study, data were collected from January 2014 to July 2016. Estimated delivery date was calculated using the average of three head circumference (HC) measurements atgestational weeks 18–20 [13]. Doppler ultrasound examinations were performed at 30 and 36 weeks of gestation, and neonatal anthropometric variables were measured at birth. Ultrasound Doppler blood flow measurements were first performed in the fasting state at 08:30 a.m. Following this examination, the women received a standard breakfast meal (SBM), comprising two slices of bread with cheese and ham, one boiled egg, one standard package with jam and one with butter, one glass of milk and one glass of juice (about 150 mL each) providing a total caloric intake of approximately 400 kcal. Ultrasound Doppler blood flow measurements were repeated at about 105 min after the meal (interquartile range of 6 min at week 30, and 11 min at week 36).

All ultrasound examinations were performed by one examiner (GLO), and lasted no more than 40 min. The same equipment was used for all participants, including an Acuson Sequoia 512 ultrasound system (Mountain View, CA, USA) with a curved transducer and a frequency bandwidth of 2–6 MHz.

MCA Doppler velocity waveforms were sampled from the proximal part of the MCA, near the circle of Willis [14]. For UA measurements, Doppler traces were sampled in a free-floating loop. Velocity was corrected for the insonation angle, which was kept as low as possible. All insonation angles were ≤26° in UA measurements, and ≤20° in MCA measurements, irrespective of gestational age. The insonation angle was ≤15° in 88% of UA examinations and in 93% of MCA examinations, irrespective of gestational age or prandial status. All Doppler measurements were performed during a period with as little fetal breathing and body movement as possible.

PI and FHR were calculated as the mean of three heart cycles. Intra-observer variations were analyzed as the intra-class correlation coefficient by means of random-effects regression, and were 0.97 for MCA-PI (n = 13) and 0.95 for UA-PI (n = 13). Changes in MCA-PI, UA-PI, and FHR were calculated by subtracting the values obtained after the meal from the fasting values. The CPR was calculated as the ratio of MCA-PI to UA-PI. Fetal biometric measurements included abdominal circumference (AC), HC, and femur length (FL). Each variable was measured three times, and the mean value was calculated. We applied z-scores of fetal biometric measurements using Norwegian growth curves [13] and birthweight [15].

### Blood sampling

After each ultrasound examination, pre- and postprandial venous blood samples were collected in sodium heparin vacutainers, kept on ice, and centrifuged within 20 min at 6°C (2500 ×*g*, 20 min). The supernatants were carefully removed and stored at ‒80°C. Glucose was measured using the hexokinase/glucose-6-phosphate dehydrogenase enzymatic in vitro test (Roche, Mannheim, Germany), performed by an accredited laboratory at the Department of Medical Biochemistry, Oslo University Hospital–Rikshospitalet.

### Statistics

Descriptive data are reported as mean and standard deviation (SD) or 95% confidence interval (CI), median and percentile, or frequency and percentage, as appropriate. Paired *t* -tests were used to compare CPR, MCA-PSV, and unadjusted MCA and UA-PI values before versus after a meal. For MCA-PSV in gestational weeks 30 and 36, we calculated multiples of the median (MoMs) according to Mari et al. [11]. Correlations were determined based on Spearman’s rank correlation. To adjust for the influence of changes in FHR, we used a linear regression model with PI value changes in the vessel of interest as the dependent variable, and FHR changes as the independent variable. With the FHR changes set as zero, the intercept value equals the corresponding change in PI value under an assumption of no change in FHR. The regression model also included a dummy variable for gestational week to compare PI changes across different gestational ages, and assess a gestational week-specific effect. All statistical tests were two-sided. A p-value of <0.05 was considered statistically significant. Data were analyzed using SPSS version 23.0 (SPSS Inc., Chicago, IL).

### Ethical approval

This study was approved by the Regional Ethics Committee (Sør-Øst 2013/1327) and performed in accordance with the standards outlines in the Helsinki Declaration. Participants gave written informed consent, and all procedures followed institutional guidelines.

## Results

The analysis included 89 participants. Table 1 presents the maternal, fetal, and neonatal characteristics.

**Table 1:** Maternal and fetal, and neonatal characteristics (n = 89).

Maternal characteristics:	Median(10^th^–90^th^ percentile)	n (%)
Maternal age, years	32 (28–40)	
Pre-pregnant body mass index	22.5 (19.5–26.4)	
Body mass index at 1st study visit	26.0 (22.7–30.6)	
Glucose level in fasting state, mmol/L, week 30	4.7 (4.4–5.2)	
Glucose level at 105 min, mmol/L, week 30	5.0 (4.0–6.2)	
Glucose level in fasting state, mmol/L, week 36	4.7 (4.2–5.1)	
Glucose level at 105 min, mmol/L, week 36	5.5 (4.4–7.3)	
Nulliparous		56 (62.9)
**Fetal and neonatal characteristics:**		
Gestational age at 1st study visit, weeks ^+ days^	30^+1^ (29^+2^–31^+0^)	
Abdominal circumference z-score, week 30	0.05 (‒0.5–0.8)	
Head circumference z-score, week 30	0.26 (‒0.7–1.1)	
Femur length z-score, week 30	0.57 (‒0.4–1.4)	
Gestational age at 2nd study visit, weeks ^+ days^	36^+1^ (35^+0^–36^+6^)	
Abdominal circumference z-score, week 36	0.09 (‒0.5–0.9)	
Head circumference z-score, week 36	0.28 (‒0.4–0.9)	
Femur length z-score, week 36	0.87 (0.2–1.5)	
Gestational age at delivery, weeks ^+ days^	40^+1^ (38^+5^–41^+5^)	
Birthweight, g	3534 (3065–4082)	
Birthweight z-score	‒0.27 (‒1.1–0.8)	
Males		43 (48.9)
Birth weight ≥ 4200 g		5 (5.7)
Birth weight < 2500 g		0
5-min Apgar score < 7		1 (1.1)

MCA-PI and UA-PI at weeks 30 and 36 were negatively correlated with FHR. These correlations were significant, except for MCA-PI in the fasting state at week 36 (Table 2).

**Table 2:** Correlations between fetal Doppler blood flow velocity variables and fetal heart rate at gestational ages of 30 and 36 weeks (n = 89).

		30 weeks			36 weeks	
	FHR in fasting state	FHR 105 min after SBM	Change in FHR	FHR in fasting state	FHR 105 min after SBM	Change in FHR
UA-PI in fasting state	r_s_ = ‒0.420 p < 0.001			r_s_ = ‒0.255 p = 0.017		
UA-PI 105 min after SBM		r_s_ = ‒0.485 p < 0.001			r_s_ = ‒0.399 p < 0.001	
Change in UA-PI			r_s_ = ‒0.582 p < 0.001			r_s_ = ‒0.449 p < 0.001
MCA-PI in fasting state	r_s_ = ‒0.227 p = 0.032			r_s_ = ‒0.140 p = 0.190		
MCA-PI 105 min after SBM		r_s_ = ‒0.344 p = 0.001			r_s_ = ‒0.342 p = 0.001	
Change in MCA-PI			r_s_ = ‒0.384 p < 0.001			r_s_ = ‒ 0.340 p = 0.001

Changes in the different variables are calculated as values after SBM minus the values before SBM.

r_s_ = Spearman’s rank correlation coefficient. UA, umbilical artery; MCA, middle cerebral artery; PI, pulsatility index; FHR, fetal heart rate; SBM, standard breakfast meal.

In week 36, FHR significantly increased after food intake (p = 0.001), measured as the mean change in beats per minute (SD) in both the MCA (+5 (13.8)) and UA (+ 5 (12.9)). Without adjustment for FHR, fetal MCA-PI decreased after the SBM in both week 30 (−0.115; p = 0.012) and week 36 (−0.255; p < 0.001). With adjustment for FHR, the MCA-PI decrease was −0.087 in week 30 (p = 0.044) and −0.194 in week 36 (p < 0.001). UA-PI did not significantly change after the SBM, with or without adjustment for FHR (Table 3).

**Table 3:** Pulsatility indices in the umbilical and middle cerebral artery before and after a meal (n = 89).

	Fasting state Mean(SD)	105 min after SBM Mean (SD)	Change Mean [95% CI]	p	r^2^
UA-PI, week 30	0.951 (0.166)	0.966 (0.161)	0.015 [−0.024, 0.054]	0.448	
UA-PI adjusted for FHR, week 30			0.019 [−0.014, 0.051]	0.253	0.32
UA-PI, week 36	0.841 (0.147)	0.808 (0.323)	−0.033 [−0.069, 0.003]	0.069	
UA-PI adjusted for FHR, week 36			−0.002 [−0.037, 0.033]	0.920	0.19
MCA-PI, week 30	2.122 (0.294)	2.006 (0.355)	−0.115 [−0.205, −0.026]	0.012	
MCA-PI adjusted for FHR, week 30			−0.087 [−0.171, −0.002]	0.044	0.14
MCA-PI, week 36	1.923 (0.375)	1.668 (0.303)	−0.255 [−0.340, −0.170]	<0.001	
MCA-PI adjusted for FHR, week 36			−0.194 [−0.276, −0.111]	<0.001	0.18

r^2^ = the proportion of the variance in the dependent variable (PI change) that is predicted from the independent variable (FHR change). PI, pulsatility index; UA, umbilical artery; MCA, middle cerebral artery; SBM, standard breakfast meal; FHR, fetal heart rate.

Changes were calculated as the value after a SBM minus the value before a SBM.

The postprandial changes in MCA-PI and UA-PI values were non-significantly larger in week 36 than in week 30. The changes at week 36 minus the changes at week 30 were −0.113 (95% CI: −0.238, 0.011; p = 0.075) for MCA-PI and −0.032 (95% CI: −0.082, 0.019; p = 0.22) for UA-PI.

**Fig 1:**
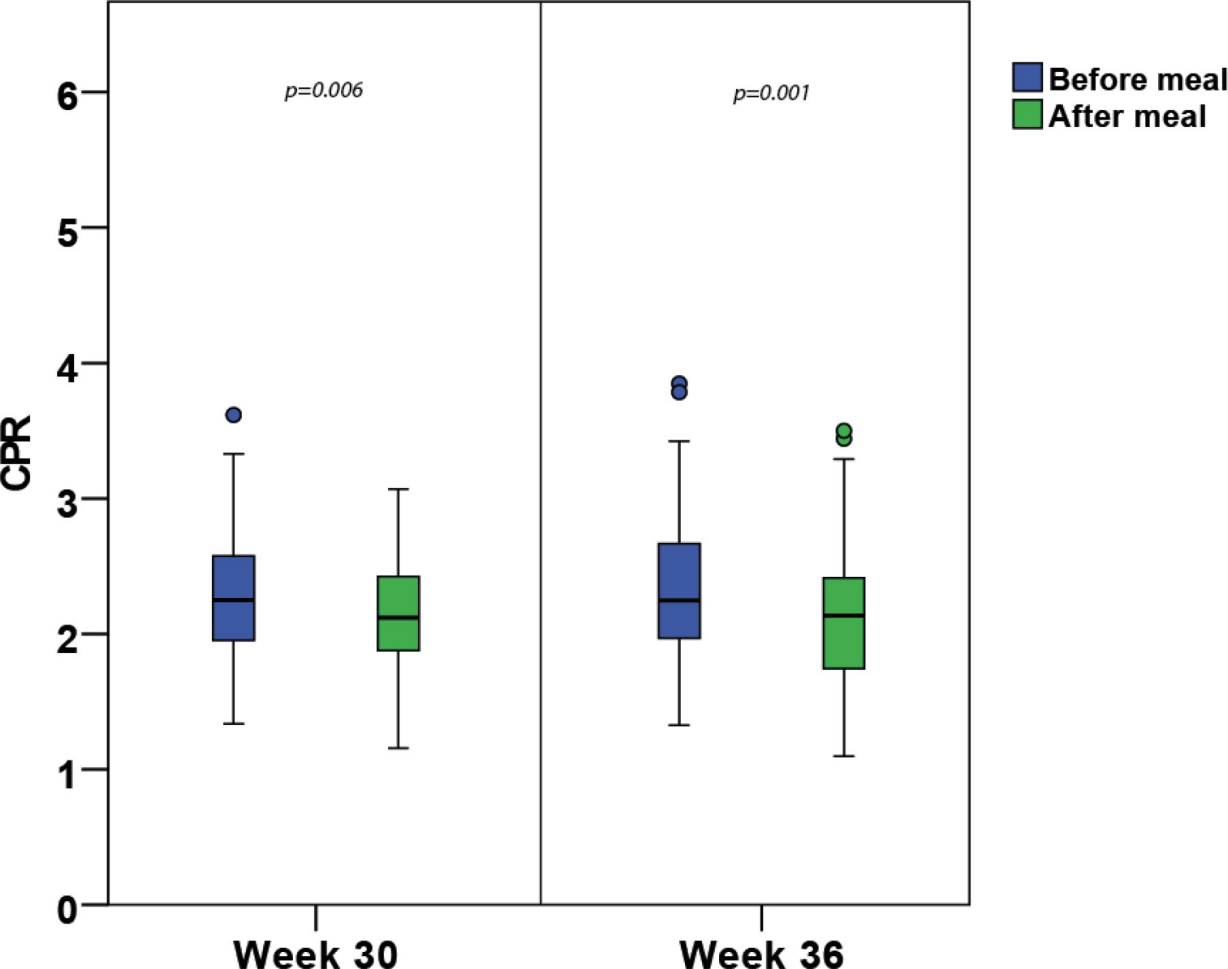
Box plot of CPR values in weeks 30 and 36. Box plot illustrating the postprandial decrease in cerebroplacental ratio (CPR) after a standard breakfast meal (SBM) in gestational weeks 30 and 36.

Using PI values unadjusted for FHR, we calculated the CPR before and after the meal at gestational weeks 30 and 36. We observed significant postprandial decrease in CPR at week 30 (−0.17; 95% CI: −0.290, −0.049; p = 0.006) and week 36 (−0.22; 95% CI: −0.355, −0.092; p = 0.001) (Fig 1). The lowest obtained single postprandial CPR value was 1.1 at week 36.

**Fig 2:**
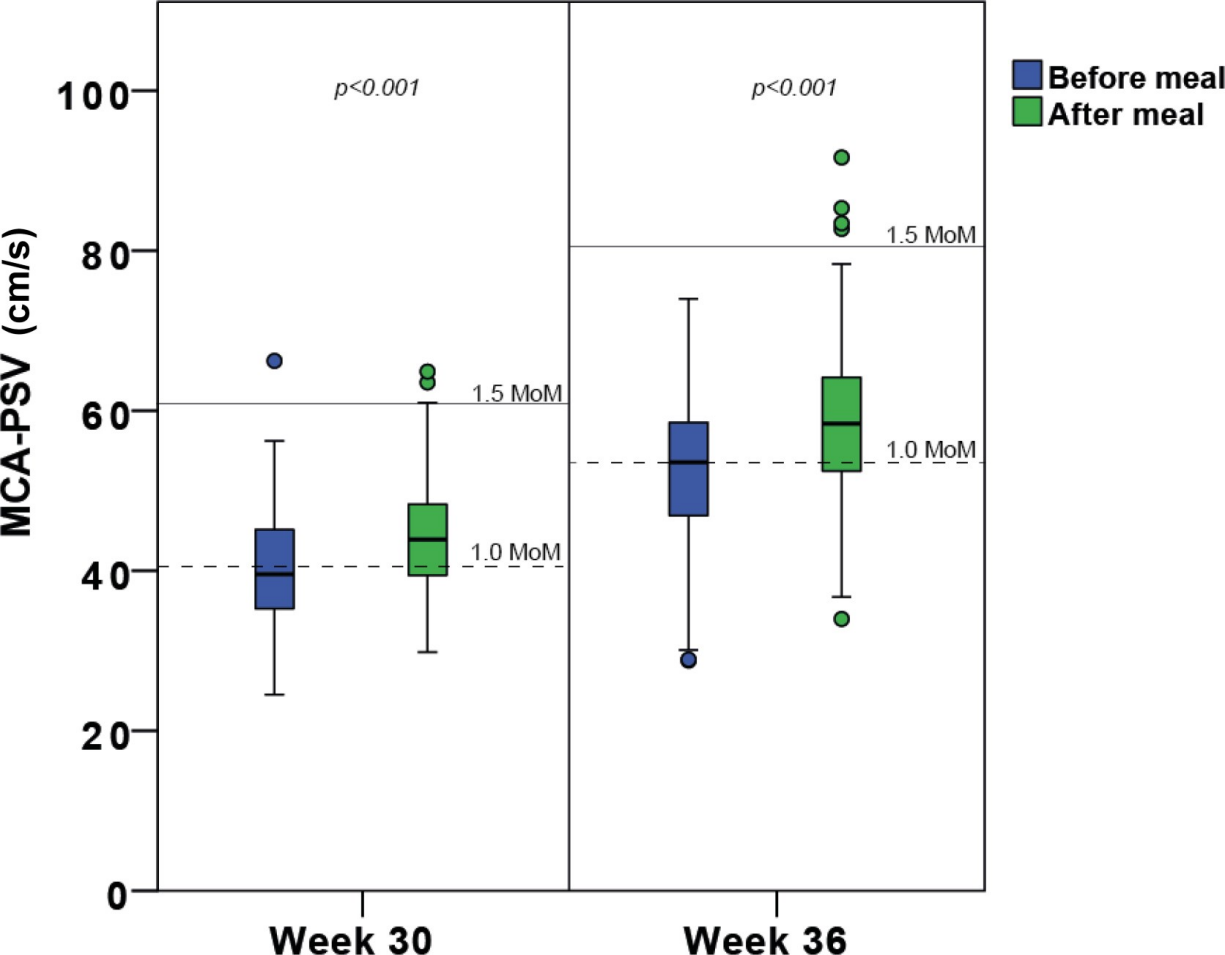
Box plot of MCA-PSV values at weeks 30 and 36. Peak systolic velocity in the middle cerebral artery (MCA-PSV) in cm/s, before and after a standard breakfast meal (SBM) in gestational weeks 30 and 36. The median values and 1.5 multiples of the median (MoM; according to Mari et al) are indicated for gestational weeks 30 and 36.

Mean MCA-PSV was significantly higher after food intake than before the SBM at week 30 (+3.9 cm/s; 95% CI: 2.32, 5.43; p < 0.001) and at week 36 (+5.9 cm/s; 95% CI: 3.81, 8.06; p < 0.001). MCA-PSV values above 1.5 MoM were found in one fasting and three postprandial fetuses in week 30, and in zero fasting and four postprandial fetuses in week 36 (Fig 2). MCA-PSV was not correlated with MCA-PI in the pre- or postprandial state (results not shown).

The postprandial changes in MCA-PI and UA-PI were not significantly correlated with maternal age; prepregnancy BMI; glucose levels before or after the meal; changes in glucose level; fetal subscapular skinfold thickness; z-scores of AC, HC, FL [13]; or birthweight [15] (r_s_< 0.208 for all variables). Postprandial MCA-PI changes did not significantly differ according to sex or parity. UA-PI was significantly increased after the meal among male fetuses in week 30 (0.054; 95% CI: 0.014, 0.094; p = 0.01) but not in week 36 (p = 0.89). UA-PI showed no significant postprandial change among female fetuses at week 30 (p = 0.38) or week 36 (p = 0.99). Postprandial CPR changes were negatively correlated with postprandial glucose concentration (r_s_ = −0.25, p = 0.031) and with change in glucose concentration (r_s_ = −0.25, p = 0.039) in week 30, but not week 36. CPR changes were positively correlated with HC z-score with borderline significance (r_s_ = 0.21, p = 0.050) in week 30 but not week 36. MCA-PSV changes in weeks 30 and 36 showed no correlation with maternal prepregnancy BMI or age.

## Discussion

Our present results showed that fetal MCA-PI significantly decreased after a standard breakfast meal in gestational weeks 30 and 36, independent of FHR. We observed no substantial postprandial changes in UA-PI, except among male fetuses in week 30. Consequently, we also observed a significant postprandial decrease in the CPR. Additionally, MCA-PSV was significantly higher after the SBM than before the meal in weeks 30 and 36.

To our knowledge, only one previous study has examined Doppler blood flow velocity variables in women (n = 19–20) before and after a meal, or before and after water intake, during the third trimester of pregnancy [16]. The authors reported a non-significant MCA-PI decrease of −0.09 following a meal, while the control group showed a non-significant MCA-PI increase of +0.03 after water intake. Two other previous studies examined normally growing fetuses (n = 15–21) in the second or third trimester, and observed decreased cerebral blood flow resistance indices after maternal glucose loading [17, 18], while another study reported increased resistance indices of the fetal carotid artery [19]. None of these four studies adjusted the Doppler indices for FHR.

Pardo et al. and Degani et al. reported increased UA vascular resistance among women with high plasma glucose levels after a glucose tolerance test [17, 19], while other studies have shown no changes in UA-PI values [18, 20, 21]. In our previous investigation of 105 individuals in gestational weeks 30–32, we also found no change in UA-PI at 2 h after oral glucose loading with 75 g [12]. In that study, we also observed a significant MCA-PI decrease after oral glucose loading (−0.22), which was greater than the decrease observed in our present study, after a standard breakfast meal (−0.087). In our present study, male fetuses showed a significant postprandial increase of UA-PI in week 30, which might be a coincidental finding.

One limitation of our present study is that the postprandial effects could not be compared to a group that was not exposed to food. Thus, we cannot be certain whether the MCA-PI decrease was part of a cyclic variation. Avian et al. recently examined 68 healthy fetuses at gestational week 36, and found that MCA-PI and UA-PI significantly decreased, while MCA-PSV significantly increased from 08.00 a.m. to 01.30 p.m. The mothers in their study were not fasting at the examinations. The authors did not adjust the data for FHR, as they did not observe any significant differences in FHR. They suggested that these variables may show diurnal variation during late gestation [22]. However, our present findings are unlikely to reflect coincidental or diurnal variations, since we observed a significant postprandial decrease in MCA-PI with no change of UA-PI, in the same fetuses at two different gestational time-points. Additionally, the time interval between the same-day examinations was shorter in our present investigation than in the study by Avian et al. Notably, all of our measurements were performed by the same examiner using the same technique, and our low intra-observer variability supports the good reliability of these measurements.

In a study of 24 normal singleton pregnancies during gestational weeks 36–40, Gillis et al. examined vascular resistance in the anterior cerebral and internal carotid arteries and umbilical arteries, pre-prandial after one night of fasting, and at 45 min and 120 min after maternal intake of 50 g glucose or of plain water. They observed significantly decreased resistance of the anterior cerebral and internal carotid arteries in the glucose group but not in the group given only water. Moreover, they reported no change in mean basal FHR in either of the two groups [21].

Sallout et al. suggested that MCA-PI in the third trimester is influenced by fetal movements based on significant increases in MCA-PSV and end diastolic velocity, reflecting increased MCA blood flow in the active state. They observed 32 fetuses in gestational weeks 30−32, and reported lower MCA-PI in active fetuses compared to inactive fetuses [23]. In our present study, FHR changes may be regarded as an indirect measure of fetal movements. We detected a significant postprandial rise in FHR in week 36, even though all measurements were taken with the participants in the most inactive possible state. As in earlier studies, we found that MCA-PI and UA-PI values were negatively correlated with FHR [24–26], and thus we adjusted the PI values accordingly. After adjustment, the postprandial MCA-PI decrease remained significant, supporting the possibility that the MCA-PI changes resulted from food intake rather than from FHR changes. Regardless of the physiological mechanisms, one should be aware of the postprandial reduction of MCA-PI when using unadjusted values in the clinical setting.

MCA-PI values according to gestational age show parabolic shape, with a gradual decline in the third trimester [14]. This decline coincides with enhanced fetal brain growth, including an almost three-fold increase in brain weight [27] and correspondingly increased energy requirements. The fetal brain vasculature exhibits substantial changes during this period, with extensive branching of the existing arterial tree, tightly coupled to structural development of the brain [1]. In this setting, the presently observed trend toward a larger decrease of MCA-PI in week 36 compared to week 30 is of physiological interest. Based on the postprandial decrease of MCA-PI, Pardo et al. suggested that fetal brain development might benefit from increasing blood flow after a meal, due to the higher blood concentrations of glucose and other nutrients [17].

Mechanisms underlying the decreased cerebral vascular resistance can only be speculated, but it is reasonable to assume that these mechanisms involve cerebral vasodilation. Vasodilatory effects may be exerted through both nervous mechanisms and circulating vasoactive substances. One possible mechanism involves the endothelium-dependent L-arginine/nitric oxide pathway [28, 29]. In human cultured umbilical vein endothelial cells, under conditions involving increased glucose levels within the normal concentration range, insulin stimulates nitric oxide production, leading to reduced vascular wall tension [30]. Thus it is interesting that the CPR decrease correlated with higher postprandial glucose values in week 30, supporting the notion that nutrition influences fetal blood flow redistribution. However, this finding was not observed in week 36.

AC z-scores were not related to the CPR and PI changes in any of the vessels in week 30 or 36. In week 30, we found a weak positive correlation between CPR changes and HC z-score, with a smaller CPR reduction related to smaller fetal HC. This might indicate that blood flow redistribution to the fetal brain was more favorable for smaller fetuses compared to larger fetuses. However, this must be cautiously interpreted. We did not find the same relationship in week 36. Moreover, we found no correlation between MCA-PI changes and HC z-scores. We speculate that our findings in this study group of presumably normally growing fetuses reflect a specific physiological postprandial effect on cerebral blood flow.

MCA-PI and UA-PI are highly relevant parameters that are used in daily clinical practice at obstetric and fetal medicine departments to assess intrauterine growth restricted fetuses (IUGR) [5]. In our present study, the MCA-PI (both unadjusted and adjusted) and CPR values were within the normal range both before and after the meal. However, our study cohort included only low-risk pregnancies. The results might be different for fetuses with a possible “brain-sparing” effect before the meal. Senoh et al. examined small-for-gestational-age fetuses, and did not observe a postprandial reduction in MCA-PI following glucose intake [18]. Thus, the presently observed postprandial reduction in MCA-PI and CPR in normal pregnancies should be acknowledged from a clinical perspective.

Fetal blood flow velocity increases as hematocrit decreases. Therefore, MCA-PSV is a clinically useful variable for revealing fetal anemia caused by maternal red blood cell alloimmunization, due to twin anemia–polycythemia sequence in monochorionic twin pregnancies, or secondary to parvovirus infection [31, 32]. The present observed physiological increase in MCA-PSV after food intake may have clinical implications with regards to the scheduling of fetal blood transfusion or delivery. In a previous study, Marie et al. found that all 111 moderately or severely anemic fetuses had MCA-PSV values above 1.5 MoM, and reported a 12% false-positive rate in 265 non-anemic fetuses when using MCA-PSV ≥1.5 MoM as the criterion [11]. Our present study did not include any neonates with known anemia at birth, and our findings indicate that a maternal meal might influence the false-positive rate for fetal anemia.

In conclusion, here we report postprandial changes in MCA-PI and CPR values, which are noteworthy for individual patient evaluations in clinical setting, although they are too moderate to be of general clinical relevance in a healthy low-risk population. The postprandial increase in MCA-PSV should be considered in the evaluation of fetal anemia. We are currently planning a further study that will include growth-restricted fetuses.
